# Expression of endoglucanases in *Pichia pastoris* under control of the GAP promoter

**DOI:** 10.1186/1475-2859-13-57

**Published:** 2014-04-18

**Authors:** Anikó Várnai, Campbell Tang, Oskar Bengtsson, Andrew Atterton, Geir Mathiesen, Vincent GH Eijsink

**Affiliations:** 1 Department of Chemistry, Biotechnology and Food Science, Norwegian University of Life Sciences, PO Box 5003Chr. Magnus Falsens vei 1, N-1432 Ås, Norway; 2 Centre for Process Innovation Limited, Wilton Centre, Wilton, Redcar TS10 4RF, UK

**Keywords:** Endoglucanase, Heterologous protein expression, *Pichia pastoris*, PichiaPink, AOX1 promoter, GAP promoter

## Abstract

**Background:**

Plant-derived biomass is a potential alternative to fossil feedstocks for a greener economy. Enzymatic saccharification of biomass has been studied extensively and endoglucanases have been found to be a prerequisite for quick initial liquefaction of biomass under industrial conditions. *Pichia pastoris*, widely used for heterologous protein expression, can be utilized for fungal endoglucanase production. The recently marketed PichiaPink™ expression system allows for rapid clone selection, and employs the methanol inducible AOX1 promoter to ensure high protein expression levels. However, methanol is toxic and poses a fire hazard, issues which become more significant at an industrial scale. It is possible to eliminate these risks and still maintain high productivity by switching to the constitutive GAP promoter.

**Results:**

In the present study, a plasmid carrying the constitutive GAP promoter was created for PichiaPink™. We then studied expression of two endoglucanases, *Af*Cel12A from *Aspergillus fumigatus* and *Ta*Cel5A from *Thermoascus aurantiacus*, regulated by either the AOX1 promoter or the GAP promoter. Initial experiments in tubes and small bioreactors showed that the levels of *Af*Cel12A obtained with the constitutive promoter were similar or higher, compared to the AOX1 promoter, whereas the levels of *Ta*Cel5A were somewhat lower. After optimization of cultivation conditions using a 15-l bioreactor, the recombinant *P. pastoris* strains utilizing the GAP promoter produced ca. 3–5 g/l of total secreted protein, with CMCase activity equivalent to 1200 nkat/ml *Af*Cel12A and 170 nkat/ml *Ta*Cel5A.

**Conclusions:**

We present a strategy for constitutive recombinant protein expression in the novel PichiaPink™ system. Both *Af*Cel12A and *Ta*Cel5A were successfully expressed constitutively in *P. pastoris* under the GAP promoter. Reasonable protein levels were reached after optimizing cultivation conditions.

## Background

Conversion of lignocellulosic biomass to energy and/or value added products provides an attractive alternative to today’s petrol-based production of chemicals and transportation fuels [1]. Saccharification of cellulose may be attained enzymatically using endoglucanases, cellobiohydrolases, lytic polysaccharide monooxygenases and β-glucosidase [2,3]. Endoglucanases (EGs) play a major role in this process, in particular in the initial liquefaction phase [4]. Production and utilization of EGs is therefore crucial for efficient biomass conversion.

The yeasts *Pichia pastoris* and *Saccharomyces cerevisiae* are frequently used hosts for heterologous production of eukaryotic proteins such as fungal biomass-degrading enzymes, mostly because of their relatively high growth rate and good productivity on inexpensive simple media [5]. In addition, yeasts are capable of necessary post-translational modifications such as glycosylation and formation of disulphide bonds. In *P. pastoris*, glycosylation of proteins is less extensive than in *S. cerevisiae*[6], which makes *P. pastoris* potentially more suitable for production of heterologous enzymes. Like *S. cerevisiae*, *P. pastoris* is able to secrete proteins, which simplifies subsequent enzyme purification processes. *P. pastoris* is known to secrete low levels of native proteins, none of which has been reported to be active on lignocellulosic biomass [7,8]. Only one intracellular β-glucosidase, an enzyme capable of monomerizing cellooligosaccharides solubilized from lignocellulosic biomass by EGs, has been identified and characterized in *P. pastoris*[9]. Hence, *P. pastoris* appears as a favorable candidate for the production of individual lignocellulose-degrading enzymes.

Over the last thirty years several promoters have been utilized for heterologous protein production in *P. pastoris*, but to date only the regulated alcohol oxidase I (AOX1) and the constitutive glyceraldehyde-3-phosphate dehydrogenase (GAP) promoters are used regularly [10-12]. The AOX1 promoter controls expression of the alcohol oxidase (AOX) in the methanol-utilization pathway. The AOX1 promoter is tightly regulated; it is repressed by glucose, glycerol and ethanol, and strongly induced by methanol [13]. The GAP promoter is constitutive, although the strength of expression varies depending on the carbon source [14]. Whilst the advantage of using the AOX1 promoter is that it allows controlled expression of heterologous proteins that may be toxic to the host, the GAP promoter simplifies cultivation by avoiding the need for toxic methanol as a carbon source.

Recently, a novel *P. pastoris* production strain PichiaPink™ has been marketed by Invitrogen (Life Technologies Corporation AS, CA, USA). PichiaPink™ is an adenine auxotrophic strain with a precise single gene deletion in the *ADE2* gene (Δ*ade2*). The absence of the *ADE2* gene leads to the accumulation of a red metabolic intermediate, phosphoribosylaminoimidazole, giving a dark pink color to the cells [15]. Transformation of *P. pastoris ade2* mutant cells with the pPink-HC vector re-introduces the knocked-out gene (*ADE2*) into the *P. pastoris* chromosome. Re-establishing the adenine biosynthetic pathway reverts the pink cell color to wild-type white, allowing visual selection of transformants [16].

In the present work, the PichiaPink™ expression system was selected for expression of two fungal endoglucanases (EGs), *Thermoascus aurantiacus Ta*Cel5A and *Aspergillus fumigatus Af*Cel12A. Plasmids carrying either the AOX1 promoter or the GAP promoter were constructed for both EGs and transformed into the double protease knock-out PichiaPink™ Strain 4. The resulting four recombinant *P. pastoris* strains were cultivated in tubes, shake flasks and bioreactors, and EG yields were compared. Finally, cultivation conditions for the best-performing recombinant *P. pastoris* strains, with the GAP promoter, were optimized for enzyme production at bioreactor scale.

## Results and discussion

### Constructing the pPink-GAP expression vectors

In the present study, we selected two EGs from different glycoside hydrolase (GH) families for expression in *P. pastoris*: a GH family 12 EG, *Af*Cel12A from *Aspergillus fumigatus* [UniProt:Q4WGT4], and a GH family 5 EG, *Ta*Cel5A from *Thermoascus aurantiacus* [UniProt:Q8TG26]. In all constructs, secretion of the enzymes was driven by the native secretion signals.

Synthetic genes encoding the two EGs were inserted downstream of the inducible AOX1 promoter into the commercially available pPink-HC vector, yielding plasmids pPink_AfCel12A and pPink_TaCel5A (Additional file 1: Figure S1). No PichiaPink™ expression vectors were available for constitutive expression of recombinant proteins. The AOX1 promoter in pPink-HC was therefore replaced with the GAP promoter and one of the two EG genes was then inserted, creating a third construct, pPink-GAP_AfCel12A (Additional file 1: Figure S1). The Kozak-sequence from the native AOX1 gene of *P. pastoris* “GAAACG” was inserted in front of the start codon to ensure efficient translation initialization [17]. A fourth construct, pPink-GAP_TaCel5A, was made by replacing the sequence encoding *Af*Cel12A with that encoding *Ta*Cel5A. The constructed plasmids were transformed into the double protease knock-out PichiaPink™ Strain 4 and integrated into the yeast chromosome, giving four recombinant *P. pastoris* strains: AOX1/AfCel12A, AOX1/TaCel5A, GAP/AfCel12A and GAP/TaCel5A.

### Screening and selection of transformants

**Figure 1:**
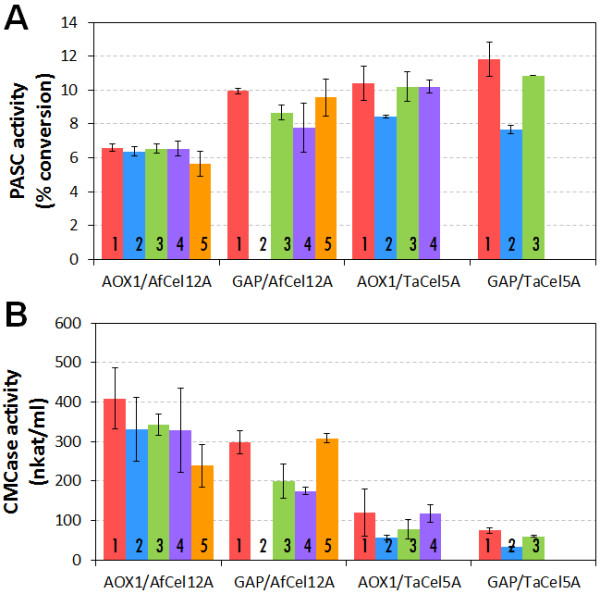
**Endoglucanase activity in culture supernatants obtained during tube-scale screening of transformants.** Culture supernatants were collected from the AOX1 strains 48 hours after induction, and from the GAP strains after 48 hours of incubation (see Methods for more details). **A)** Endoglucanase activity was measured on filter paper by incubating 10 μl culture supernatant with 40 μl of a suspension of phosphoric acid swollen filter paper (1% w/v) and 10 μl 50 mM Na-citrate buffer (pH 5.0) for 1 hour at 50°C. **B)** Endoglucanase activity was measured on carboxymethylcellulose by incubating 50 μl appropriately diluted culture supernatant with 450 μl 1% (w/v) carboxymethylcellulose in 50 mM Na-citrate buffer (pH 5.0) for 10 min at 50°C. Enzyme activities were assessed by measuring formation of reducing sugars. Filter paper activity is expressed as percentage of substrate conversion; CMCase activity is expressed as nkat/ml. The activity values were background corrected by measuring reducing sugars present in the culture supernatants. Since culture supernatants of PichiaPink^TM^ transformed with the empty vector, lacking endoglucanases, showed no activity, all activity can be attributed to the recombinant endoglucanases. The data are means from triplicate experiments; error bars reflect standard deviations. The clone number is indicated in the bars.

Three to five white colonies from each transformation were selected for initial screening. With the exception of one clone, GAP/TaCel5A clone 2, all clones formed uniformly white colonies after being re-streaked on fresh PAD selection plates. The GAP/TaCel5A clone 2 transformant gave sectored (both white and pink) colonies, which indicate unstable integration of the vector into the *P. pastoris* genome [16]. For most of the tested clones, both enzymes were successfully produced and secreted, and enzymatic activity could be detected in culture supernatants (Figure 1 and Additional file 1: Figure S2). The GAP/AfCel12A clone 2 did not produce detectable amounts of enzyme, formed colonies with different morphology, and grew poorly on YPD medium as compared to *P. pastoris*. This clone was probably a contaminating strain able to grow due to the lack of antibiotics in the medium and was excluded from further work.

Heterologous expression in both *P. pastoris* and *S. cerevisiae* may lead to hyperglycosylation, which is attributed primarily to N-glycosylation [18]. Glycosylation may lead to the recombinant proteins appearing at unexpected positions in SDS-PAGE gels (1–2 kDa shift per glycosylation), whereas heterogeneous hyperglycosylation may yield proteins appearing as smears [18]. To assess N-glycosylation, one clone of each transformant was selected and the proteins in the cell-free culture supernatants were subjected to EndoH treatment. Protein bands appeared on the SDS-PAGE gel of the cell-free supernatants at the expected molecular weights, 24 kDa for *Af*Cel12A and 37 kDa for *Ta*Cel5A (Additional file 1: Figure S2), indicating that the proteins were processed correctly. The EndoH treatment did not change the apparent molecular weight of the secreted EGs, confirming that the enzymes were produced without excessive N-glycosylation (Additional file 1: Figure S3), which is in agreement with both enzymes having a limited number of putative N-glycosylation sites.

EG activity was measured in supernatants from the recombinant *P. pastoris* clones using a cellulosic model substrate (Phosphoric Acid Swollen Cellulose from Filter Paper, Whatman No. 1, PASC-FP) or carboxymethylcellulose (CMC), a water-soluble cellulose derivative. Supernatants from all checked clones exhibited activity on PASC-FP and CMC (Figure 1; note that the activity levels on CMC seem proportional to band intensities in Additional file 1: Figure S2; this is not the case for the activities on PASC-FP due to the non-linearity of the PASC-FP assay). Figure 1 shows that enzyme levels produced with the GAP and AOX1 promoters are similar, with some variations that are explored further below. A control strain transformed with the vector pPink-HC showed no endoglucanase activity on PASC-FP, confirming that the activity in the supernatants of the production strains was due to secreted recombinant EG.

Two clones from each transformation (with the highest activity in Figure 1) were evaluated in shake flask cultures. Generally, enzyme activity reached levels similar to those observed during the tube scale screening, confirming that EG activity varies slightly with the promoter used. The activity of both enzymes reached higher levels on PASC-FP when expressed under the constitutive GAP promoter than under the AOX1 promoter, in particular for *Af*Cel12A, which is in accordance with Figure 1A.

### Fed-batch cultivation in bioreactors

**Figure 2:**
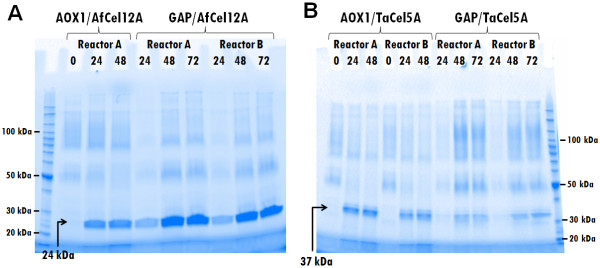
**Endoglucanase production in 3-liter bioreactors with fed-batch cultivation.** One clone of each PichiaPink™ recombinant strain was grown in two parallel 3-l fermenters. The SDS-PAGE figures show the accumulation of **A)***Af*Cel12A and **B)***Ta*Cel5A. Sample lanes are marked with the age of the culture in hours for each fermenter. Reactor B of AOX1/AfCel12A is missing from the gel as cells stopped growing in the batch phase. The bands appearing at approx. 24 kDa (panel A) represent *Af*Cel12A; the bands appearing at approx. 37 kDa represent *Ta*Cel5A (panel B).

To reach reasonable production of the endoglucanases, clones giving the highest enzyme titers were selected for each transformant, namely: AOX1/AfCel12A clone 1, GAP/AfCel12A clone 1, AOX1/TaCel5A clone 1 and GAP/TaCel5A clone 1. Firstly, these clones were cultivated in a 3-l bioreactor using fed-batch cultivation (1 l initial volume in batch phase) following the protocol described by Stratton et al. [19] without optimizing parameters of cultivation. The levels of secreted total proteins varied between 1–5 g/l, which is common for the production of lignocellulolytic enzymes in yeast in fed-batch cultivations [20]. Enzyme expression, monitored with SDS-PAGE (Figure 2), was clearly higher than that in the tube-scale cultures used in the initial screening phase (Additional file 1: Figure S2). Accordingly, the endoglucanase activity in the supernatants obtained from the bioreactors amounted to 10.0% (AOX1/AfCel12A), 11.7% (GAP/AfCel12A), 13.6% (AOX1/TaCel5A) and 14.2% of conversion (GAP/TaCel5A) of PASC-FP in the filter paper assay, and to 630 nkat/ml (AOX1/AfCel12A), 1200 nkat/ml (GAP/AfCel12A), 290 nkat/ml (AOX1/TaCel5A) and 190 nkat/ml (GAP/TaCel5A) on CMC, which are values higher than those obtained in the initial screening experiments (Figure 1). Whilst *Af*Cel12A appeared as the major band on the gel, with the GAP promoter working better, bands representing *Ta*Cel5A were less prominent and in this case the AOX1 promoter worked slightly better (Figure 2).

*Af*Cel12A and *Ta*Cel5A were purified from a culture supernatant and their specific activities on CMC were determined to be 1040 and 1450 nkat/mg, respectively. Comparing these values with specific activities measured for culture supernatants of the GAP promoter indicated that *Af*Cel12A amounted to approximately 35% of total protein in the best supernatants shown in Figure 2, whereas this value was approximately 5% for *Ta*Cel5A. The ratio of endoglucanase to background proteins was higher in case of methanol-induced expression, *Af*Cel12A and *Ta*Cel5A making up approximately 60% and 25% of total secreted protein, respectively. Thus, in terms of the amount of EG protein produced, production of *Af*Cel12A was more successful, similar to what was observed in the experiments with tubes and shake flasks.

Fermentation development in 15-l stainless steel steam-in-place fermenters was carried out only for the GAP strains expressing *Af*Cel12A and *Ta*Cel5A, since the screening and initial fermentation experiments consistently indicated that the GAP promoter worked better than (*Af*Cel12A) or approximately as well as (*Ta*Cel5A) the AOX1 promoter. Fermentation development in such fermenters facilitates straightforward upscaling to pilot plant scale production and beyond. Cultivation conditions were optimized with particular focus on temperature, carbon source and feeding regime. Initially, both glycerol and glucose were evaluated, and feeding regimes were set based on the fermentation guidelines by Stratton et al. [19].

Firstly, the effect of increasing the glycerol feed rate was tested. Increasing the feed from 1714 g to 2470 g over 70 hours resulted in increased biomass and total secreted protein with both the GAP/TaCel5A and GAP/AfCel12A strains, as would be expected from the extra carbon availability (Table 1). The overall secreted protein almost doubled with the feed increase (from 2.8 to 3.8 g/l) for the *Ta*Cel5A expressing strain, whereas no significant increase was observed (from 3.4 to 3.3 g/l) for the strain expressing *Af*Cel12A. However, there was a marked difference between the strains in terms of enhanced endoglucanase productivity as judged by band intensities on the SDS-PAGE gels. Whilst increased glycerol feed led to no improvement of the *Ta*Cel5A yield, there was a significant increase in *Af*Cel12A yield as well as in its proportion relative to other secreted proteins (Additional file 1: Figure S4).

**Table 1:** **Optimization of fed-batch cultivation of recombinant PichiaPink™ strains expressing *Af*** **Cel12A and *Ta*** **Cel5A endoglucanases**

**Run**	**Strain**	**Temperature (°C)**	**Feed**	**Feed regime**	**Max dry cell weight (g/l)**	**Total supernatant protein (g/l)**
1	**GAP/TaCel5A**	30	Glycerol	1714 g over 70 h	109	2.8
2^b^	**GAP/TaCel5A**	30	Glycerol	2470 g over 70 h	155 ± 5	3.8 ± 0.4
3	**GAP/TaCel5A**	30	Glucose	2470 g over 70 h	145	4.9
4	**GAP/TaCel5A**	25	Glycerol	2470 g over 70 h	155	5.4
5	**GAP/AfCel12A**	30	Glycerol	1714 g over 70 h	132	3.4
6^b^	**GAP/AfCel12A**	30	Glycerol	2470 g over 70 h	146 ± 9	3.3 ± 0.3
7	**GAP/AfCel12A**	30	Glucose	2470 g over 70 h	100	n.d.^a^
8	**GAP/AfCel12A**	25	Glycerol	2470 g over 70 h	170	7.3

^a^Not determined; ^b^Optimal parameters, fermentations run in 5 parallels.

**Figure 3:**
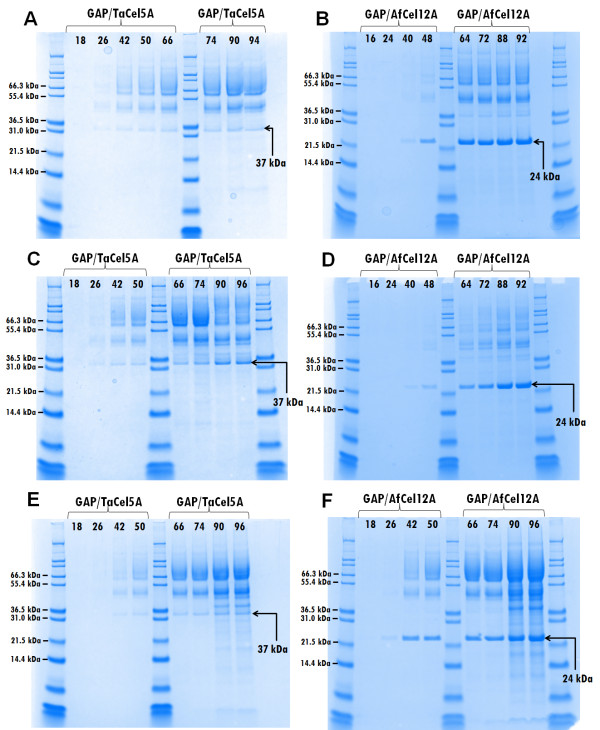
**Optimization of fed-batch cultivations for production of *Ta*Cel5A and *Af*Cel12A.** Fed-batch cultivations for producing *Ta*Cel5A **(A, C, E)** and *Af*Cel12A **(B, D, F)** were performed in glycerol at 30°C **(A, B)**, in glucose at 30°C **(C, D)** and in glycerol at 25°C **(E, F)**. Carbon sources were introduced as a stepped feed of 2470 g (3.9 l) of glycerol or glucose over 70 h, following a 24-h batch phase, with an initial volume of 5 l. Fermentation parameters were controlled at standard settings (see Methods). Culture supernatant samples were collected at various time points and analyzed by SDS-PAGE (15 μl sample size). Sample lanes are marked with the age of the culture in hours. Bands marked as 37 kDa represent *Ta*Cel5A. Bands marked as 24 kDa represent *Af*Cel12A. The protein standard was Mark12 Unstained Standard (Invitrogen). Additional data for these fermentations are provided in Table 1.

Fermentations were carried out to analyze the effect of temperature, increased glycerol feed and carbon source on the GAP/TaCel5A strain and the GAP/AfCel12A strain. The PichiaPink™ cells were grown in 15-l scale bioreactors with a 5-l initial volume for 24 hours in batch mode, followed by feeding as indicated; see Methods for more details. Runs were performed as single experiments; however, optimized runs were performed five times, and standard deviations are provided. SDS-PAGE analyses of the culture supernatants are shown in Figure 3.

Secondly, glycerol and glucose were compared as the carbon source in the fed-batch phase. For both strains, glycerol appeared to be a better carbon source than glucose, resulting in higher biomass concentrations, and higher endoglucanase yields (Table 1 and Figure 3). Additionally, the glycerol feed resulted in faster *Af*Cel12A production, with maximum levels being reached earlier during the fermentation (compare Figure 3B and D). Previous studies on comparing glucose and glycerol in GAP-driven expression have given varying results. For example, glycerol gave better yields for human angiostatin [21] and a phytase [22], whilst glucose gave better yields in other cases [14,23]. The choice of carbon source thus appears to depend on the individual gene. Our results indicate that glycerol would be a better carbon source to use in the case of *Ta*Cel5A and *Af*Cel12A.

Thirdly, the effect of lowering the temperature on growth and enzyme production was evaluated. Decreasing the cultivation temperature from 30°C to 25°C hardly affected growth and supernatant protein concentrations for the *Ta*Cel5A clone (Table 1 and Figure 3) but the SDS-PAGE analyses indicated slightly higher relative yields of *Ta*Cel5A at 30°C (Figure 3A and E). On the other hand, total secreted protein by *P. pastoris* expressing *Af*Cel12A was considerably higher at 25°C (7.3 g/l at 25°C compared to 3.8 g/l at 30°C) (Table 1). The SDS-PAGE analyses (Figure 3B and F) show that maximum *Af*Cel12A levels were similar at both temperatures and clearly show that the EG produced at 30°C contained much less contaminating proteins.

All in all, under optimized conditions we were able to reach protein concentrations in the supernatants in the range of 5 g/l. CMCase activity in the supernatants of the optimized cultures (grown on glycerol at 30°C) amounted to 1200 ± 100 nkat/ml (GAP/AfCel12A) and 170 ± 10 (GAP/TaCel5A), representing a two- to three-fold increase compared to the shake flask cultivations. Figure 3 (panels A and B) shows that under these optimized conditions, recombinant *Af*Cel12A is the major extracellular protein, whereas *Ta*Cel5A is produced at considerably lower levels. By comparing the specific CMCase activity in the culture supernatants to the specific CMCase activity of the purified EGs, *Af*Cel12A was estimated to represent 36% of total secreted protein, whereas this value was 4% for *Ta*Cel5A.

As to comparing the GAP and AOX1 promoters, the data show that the overall efficiency of protein expression under the regulation of AOX1 or GAP promoters in *P. pastoris* depends on the protein that is expressed. This is also reflected in existing literature data [12]. In several cases, the GAP promoter was found to be superior to the AOX1 promoter [14,24], whereas in other cases the picture was less clear [20].

As far as the downstream processing is concerned, recombinant endoglucanase preparations may not need to be purified prior to their application in biomass valorization for a number of reasons. Other secreted proteins may act as surfactants and reduce non-productive binding of cellulases [25]. In addition, as the currently used PichiaPink™ strain 4 is double protease deficient, proteolysis of the endoglucanase is unlikely. In industrial applications, where processes are lengthier than in the laboratory, this is important as it would prolong the durability of the EGs. Furthermore, *P. pastoris* strains, in general, produce no lignocellulolytic enzymes [7,8]. Some intracellular β-glucosidase [9] may be released to the cell-free culture broth as a result of cell lysis, but this would rather enhance than retard lignocellulose hydrolysis. Hence, *P. pastoris* appears as a favorable candidate for production of endoglucanases and other lignocellulose-degrading enzymes.

## Conclusions

We have constructed a plasmid (pPink-GAP) for constitutive expression of recombinant proteins with the PichiaPink™ system in order to simplify enzyme production compared to methanol inducible expression systems. Two endoglucanases, *Af*Cel12A from *Aspergillus fumigatus* and *Ta*Cel5A from *Thermoascus aurantiacus*, were successfully expressed in PichiaPink™ under the GAP promoter, and transformants produced up to 5 g/l total secreted protein after optimization in bioreactors, with 36% and 4% of the total proteins being *Af*Cel12A and *Ta*Cel5A, respectively. We found that increasing the glycerol feed resulted in higher endoglucanase yields, that glycerol was more favorable as a carbon source than glucose, and that cultivating transformants at 30°C led to a smaller proportion of background proteins relative to the endoglucanase than at 25°C. Due to the limited number of potential N-glycosylation sites (none and one, respectively), *Af*Cel12A and *Ta*Cel5A both could be produced without hyperglycosylation, simplifying downstream processing.

## Methods

### Bacterial strains and plasmids

Chemically competent *E. coli* TOP10 (Invitrogen, Life Technologies Corporation AS, CA, USA) was used as subcloning host for the pPink-HC plasmids. The PichiaPink™ Secretion Optimization Kit, containing PichiaPink™ Strain 4 (*P. pastoris ade2*, *prb1*, *pep4*–adenine auxotroph and double protease knock-out strain), the expression vector pPink-HC and PichiaPink™ Media Kit, as well as the pGAPZα A vector were all purchased from Invitrogen.

The coding regions, including the native signal sequence, of the genes *cel12A* of *Aspergillus fumigatus* encoding *Af*Cel12A [GenBank:NC_007200.1 REGION: 1511096..1511900, UniProt:Q4WGT4] and *eg1* of *Thermoascus aurantiacus* encoding *Ta*Cel5A [GenBank:AF487830.2, UniProt:Q8TG26] were synthesized by GenScript USA Inc. (NJ, USA) using standard procedures for codon optimization for *Pichia* expression. The Kozak-sequence from the native AOX1 gene of *P. pastoris* (GAAACG) was inserted before the start codon (Additional file 1: Figure S1D) to enhance translation initiation [17]. All primers were synthesized by Eurofins MWG Synthesis GmbH (Germany) and the sequences of all PCR-amplified DNA fragments were confirmed by GATC Biotech AG (Germany).

### Media and chemicals

*E. coli* was grown on Brain-Heart-Infusion medium (Lonza, ME, USA) with 100 μg/ml ampicillin (Sigma-Aldrich, Inc, St. Louis, MO). Agar plates were solidified with 1.6% (w/v) BactoAgar (Saveen Werner AB, Sweden). For *P. pastoris*, the PichiaPink™ Media Kit (Invitrogen) including BMGY, BMMY, YPD and YPDS media and YPD and PAD (*Pichia* adenine dropout) plates was used for cultivation. All chemicals for the medium used in the bioreactors were purchased from Sigma-Aldrich unless otherwise stated.

### Plasmid construction and transformation of P. pastoris

To insert the endoglucanase genes into pPink-HC with the AOX1 promoter, the synthetic *cel12A* and *eg1* genes were excised from the pUC57 vector using *Acc*65I and *Eco*RI (both from New England BioLabs [NEB] Inc, MA) and ligated into the same sites of pPink-HC, yielding pPink_AfCel12A and pPink_TaCel5A, respectively (Additional file 1: Figure S1A).

In order to replace the methanol-inducible AOX1 promoter with the constitutive GAP promoter, we first amplified the GAP promoter from the pGAPZα (Invitrogen) plasmid with primers P1 (CAGTGAATTGAGATCTTTTTTGTAGAAATGTCTTGGTGTCC) and P2 (GTCTTCATCGTTTC*GAATTC***CGTTTC**GAAATAGTTGTTCAATTGAT) and the *cel12A* gene from the pPink_AfCel12A plasmid with primers P3 (CCCTATTTCAATCAATTGAACAACTATTTC**GAAACG***GAATTC*GAAACGAT) and P4 (ATGGCCGGCCGGTAC) using Phusion high-fidelity DNA polymerase (NEB); in P2 and P3 the Kozak sequence is indicated in bold and the *Eco*RI site in italic. These two PCR fragments with 56 overlapping base pairs were fused together in a splicing by overlap extension-PCR (SOE-PCR) reaction [26] using primers P1 and P4 (Additional file 1: Figure S1B). The resulting fragment was subsequently In-Fusion cloned into the *Bgl*II/*Acc*65I fragment of the pPink_AfCel12A, resulting in pPink-GAP_AfCel12A (Additional file 1: Figure S1C). This strategy led to the introduction of an *Eco*RI site after the GAP promoter (encoded by primer P2), allowing exchange of the target gene. The fourth plasmid was constructed by replacing the synthesized *cel12A* gene in the vector pPink-GAP_AfCel12A with the synthesized *eg1* gene using *Eco*RI and *Acc*65I, yielding pPink-GAP_TaCel5A.

The four vectors were linearized with *Afl*II (NEB) and 3 μg of each plasmid were transformed into electrocompetent cells of *P. pastoris* PichiaPink™ Strain 4. The PichiaPink™ strain was made electrocompetent by following the manufacturer’s instruction [27] and transformed using a Bio-Rad Gene Pulser II electroporation system (Bio-Rad Laboratories, CA, USA) at 1.8 kV, 25 μF, 200 Ω, resulting in a time constant of 5 ms. After transformation the cells were incubated in YPDS media overnight and then spread on *Pichia* adenine dropout (PAD) selection plates (Invitrogen) for 8–12 days at 30°C until colonies formed. Of the colonies, 3–5 white ones were picked and re-streaked on fresh PAD plates, glycerol stocks were prepared from overnight cultures in YPD medium in 20% (v/v) glycerol. Transformants were named based on the promoter/gene combination as *P. pastoris* AOX1/AfCel12A, AOX1/TaCel5A, GAP/AfCel12A and GAP/TaCel5A.

### Cultivation of PichiaPink™ transformants

Cellulase production by recombinant *P. pastoris* strains was first tested at tube scale. To do so, 10 ml medium was incubated in 50-ml Falcon tubes (30°C, shaken at 160 rpm, at least duplicates). The AOX1 strains (*P. pastoris* AOX1/AfCel12A and AOX1/TaCel5A) were grown for 48 hours in BMGY medium (containing 1% (v/v) glycerol) to increase cell density before induction. After 48 hours the cells were pelleted by centrifugation (1500 g for 5 min) and resuspended in 1 ml BMMY medium (containing 0.5% (v/v) methanol), and further incubated for 24 hours (the total volume of the culture was approx. 4 ml). Then a sample of 100 μl culture was taken out, 100 μl 40% (v/v) methanol was added and the cultures were incubated for another 24 hours. Cell-free culture supernatants were harvested by centrifugation (1500 g for 10 min) 48 hours after induction and cellulase activity was measured (see below). The PichiaPink™ GAP strains (GAP/AfCel12A and GAP/TaCel5A) were grown for 72 hours in 10 ml YPD medium in 50-ml Falcon tubes at 30°C shaken at 160 rpm, 1 ml sample was collected every 24 hours. Cell growth was determined by measuring the optical density of the cultures at 600 nm (OD_600_). Samples were centrifuged at 13 000 g for 1 min and the cell-free supernatants were assayed for cellulase activity.

In the second evaluation, *P. pastoris* transformants were cultivated in 250 ml baffled shake flasks containing 10 ml BMGY (AOX1 strains) or YPD medium (GAP strains) in triplicates, at 30°C, with shaking at 220 rpm. The starting OD_600_ was set to 0.2 with overnight inoculums grown in YPD medium. The cultivation and sampling were similar to the tube scale experiment (see above) with the exception that PichiaPink™ clones with AOX1 constructs were grown on BMGY medium for only 24 hours prior to induction. Cell growth and cellulase activity in supernatants were continuously monitored throughout the cultivation.

Next, four *P. pastoris* clones (AOX1/AfCel12A-1, AOX1/TaCel5A-1, GAP/AfCel12A-1 and GAP/TaCel5A-1) were cultivated in 3-l bioreactors (Applikon, The Netherlands) in fed-batch mode according to Stratton et al. [19]. The clones were pre-cultured in 100 ml MGY medium in 1-l baffled shake flasks. One liter basal salts medium supplemented with 2 ml/l PTM1 trace salt solution [19] was inoculated with 30–50 ml pre-culture, giving a starting OD_600_ of 1.0–1.5. Cells were grown until depletion of glycerol, then 100% methanol (AOX1 clones) or 55% (w/v) glucose (GAP clones) both containing 0.2% (v/v) PTM1 solution was fed into the reactors. The methanol feed rate was initially set to 2 ml/h and was periodically halted to observe dissolved oxygen spikes according to Stratton et al. [19], then slowly increased to 6 ml/h, keeping the dissolved oxygen level at 35% as suggested by Stratton et al. [19]. The glucose feed rate was 10 ml/h but was periodically halted until all residual glucose was consumed, leading to variations in the total amount of glucose fed. Samples were taken at regular intervals to follow cell growth and enzyme production; the cultures were harvested after 72 hours of total fermentation time. AOX1/AfCel12A and AOX1/TaCel5A strains were fed in total 130 g and 280 g MeOH, respectively; GAP/AfCel12A was fed in total 330 g (Reactor A) or 280 g (Reactor B) glucose, and GAP/TaCel5A 250 g (Reactor A) and 300 g (Reactor B) glucose.

The GAP clones were selected for further optimization. Fermentations were carried out in 15-l stainless steel steam-in-place bioreactors (Sartorius Biostat B). Batch phase cultivations were carried out in basal salts medium containing additionally 0.5 ml polypropylene glycol P2000 and 0.025 g CuSO_4_.5H_2_O per liter [19]. Basal salts medium was supplemented with 4.35 ml/l PTM1 trace metals solution [27]. Fermenters were set up with 5 l of Basal Salts Medium, and the PTM1 trace metals solution added after sterilization via a 0.2 μm sterile filter. Each fermenter was inoculated with 2% v/v of selected *P. pastoris* strain grown for 24 hours (up to OD_600_ of 15–30) on YPG (40 g/l glycerol, 20 g/l soy peptone, 10 g/l yeast extract) at 30°C, 220 rpm. When carbon limitation (monitored by dissolved oxygen level) became noticeable (after approximately 24 hours), the selected feed strategy was applied. Glycerol and glucose feedstocks consisted of 12 ml/l PTM1 salt solution with 630 g/l glycerol or glucose, respectively. The feeding strategy used is detailed as follows (cumulative feed in bold): 0–24 h = 0 g/h **(0 g)**; 24–32 h = 12.6 g/h **(101 g)**; 32–40 h = 18.9 g/h **(252 g)**; 40–48 h = 25.2 g/h **(454 g)**; 48–56 h = 31.5 g/h **(706 g)**; 56–64 h = 37.8 g/h **(1008 g)**; 64–72 h = 44.1 g/h **(1361 g)**; 72–94 h = 50.4 g/h **(2470 g)**. Unless stated otherwise in the results section, settings for fermentations were 30°C, pH 5.0 (maintained by 12% (w/w) ammonium hydroxide). Aeration was set at 5 l/min (1 vvm) initial flow, with initial agitation at 250 rpm, followed by cascading to maintain 30% dissolved O_2_. After 24 hours, the airflow was increased to 7.5 l/min and after 48 hours to 10 l/min, with aeration always cascaded to maintain 30% dissolved O_2_. Dry cell weights were determined by centrifuging 10 ml samples, discarding the supernatant, and drying the pellet at 70°C until a constant weight was obtained.

### Analysis of endoglucanase production

Protein expression was analyzed with SDS-PAGE gel electrophoresis of cell-free supernatants. During selection we used the Mini-Protean TGX Stain Free Gel system (Bio-Rad Laboratories, Inc., CA) with 10% precast polyacrylamide gels following the manufacturer’s instructions; the proteins were visualized with a Bio-Rad Criterion Stain Free Imager system (Bio-Rad Laboratories). During fermentation optimization, SDS-PAGE gel electrophoresis of cell-free supernatants was carried out using the Novex X-Cell II Mini-Cell gel system (Invitrogen) with NuPAGE 10% Bis-Tris gels (Invitrogen Cat. No. NP0302BOX) and MES SDS Running Buffer (Invitrogen Cat. No. NP0002). Gels were stained with Coomassie Blue.

Protein concentrations were determined using the Bicinchoninic Acid Protein Assay Kit (Cat. No. BCA1-1KT, Sigma-Aldrich). BSA was used for preparation of the standard curve.

Cellulase activity was measured in 60 μl reaction mixtures by incubating 10 μl cell-free supernatants with phosphoric acid-swollen cellulose from Whatman No 1 filter paper (PASC-FP) at 6.7 g/l final substrate concentration in 50 mM Na-citrate buffer (pH 5.0) at 50°C for 60 min. Enzyme activities are expressed as a percentage of the substrate converted, calculated as the ratio of released reducing sugars to the initial substrate concentration, multiplied by a hydrolysis factor of 0.9. The concentration of reducing sugars was measured using 3,5-dinitrosalicylic acid reagent [28].

The endoglucanase activity was also assayed on carboxymethylcellulose (CMC) as CMCase activity based on the assay by the International Union of Pure and Applied Chemistry [29]. Cell-free supernatants or purified enzyme solutions were diluted to reach the linear range of the assay, then 50 μl appropriately diluted enzyme samples were incubated in 500 μl reaction mixtures with CMC at 9 g/l final substrate concentration in 50 mM Na-citrate buffer (pH 5.0) at 50°C for 10 min. Enzyme activities are expressed as katals, and calculated as the ratio of released reducing sugars to incubation time. The concentration of reducing sugars was measured with 3,5-dinitrosalicylic acid reagent [28].

### Purification of endoglucanase

Endoglucanases *Ta*Cel5A and *Af*Cel12A were purified from supernatants of the 3-l bioreactors. Culture broths were concentrated with Vivaflow 50 tangential crossflow concentrator (MWCO 10000 Da, Sartorius Stedim Biotech GmbH, Germany). Concentrated supernatants were thoroughly washed with ultrapure water to remove residual salts remaining from the culture media, after which the water was exchanged to the equilibration buffer of the subsequent purification step. *Ta*Cel5A was loaded on a 5-ml DEAE purification column (GE Healthcare Bio-Sciences AB, Sweden) equilibrated with 20 mM Na-acetate buffer (pH set to 5.0), and eluted by increasing the NaCl-concentration from 0 to 0.15 M linearly over 6 column volumes. Protein fractions eluting in 0.12–0.15 M NaCl concentration were collected. *Af*Cel12A was purified on a 5-ml SP-FF purification column (GE Healthcare Bio-Sciences AB, Sweden) equilibrated with 20 mM Na-citrate buffer (pH set to 3.0), and eluted with a stepwise gradient increasing NaCl concentration from 0 M to 0.10 and then further to 0.13 M. The protein fractions eluting in 0.10–0.13 M NaCl concentration were collected. The enzyme solution was concentrated by ultrafiltration with 10 kDa MWCO Vivaspin 20 ml tubes (Sartorius Stedim Biotech GmbH, Germany), and thoroughly washed with the equilibration buffer of the preceding purification step to remove NaCl. Protein purity was verified using SDS-PAGE and estimated to be over 95% for both enzymes.

## Abbreviations

AOX1: Alcohol oxidase I; EG: Endoglucanase; GAP: Glyceraldehyde-3-phosphate dehydrogenase; GH: Glycoside hydrolase; OD: Optical density; PASC-FP: Phosphoric acid swollen cellulose from filter paper; SDS-PAGE: Sodium dodecyl sulfate polyacrylamide gel electrophoresis.

## Competing interests

The authors declare no competing interest.

## Authors’ contributions

AV, CT, GM and VE designed and coordinated the work. AV, CT, OB, AA GM carried out the experiments. AV, CT, GM and VE wrote the manuscript. All authors participated in finalizing the manuscript and have read and approved the final manuscript.

## Supplementary Material

Additional file 1 Supplementary figures, containing Figures S1-S4. Click here for file
