# Supplementary material for: Expression of endoglucanases in Pichia pastoris under control of the GAP promoter
Source: Microb Cell Fact. 2014 Apr 18;13:57. doi: 10.1186/1475-2859-13-57 (PMC4021498; doi:10.1186/1475-2859-13-57)
Supplement: Additional file 1 — Supplementary figures, containing Figures S1-S4. [file 1475-2859-13-57-S1.pdf]

Supplementary figures

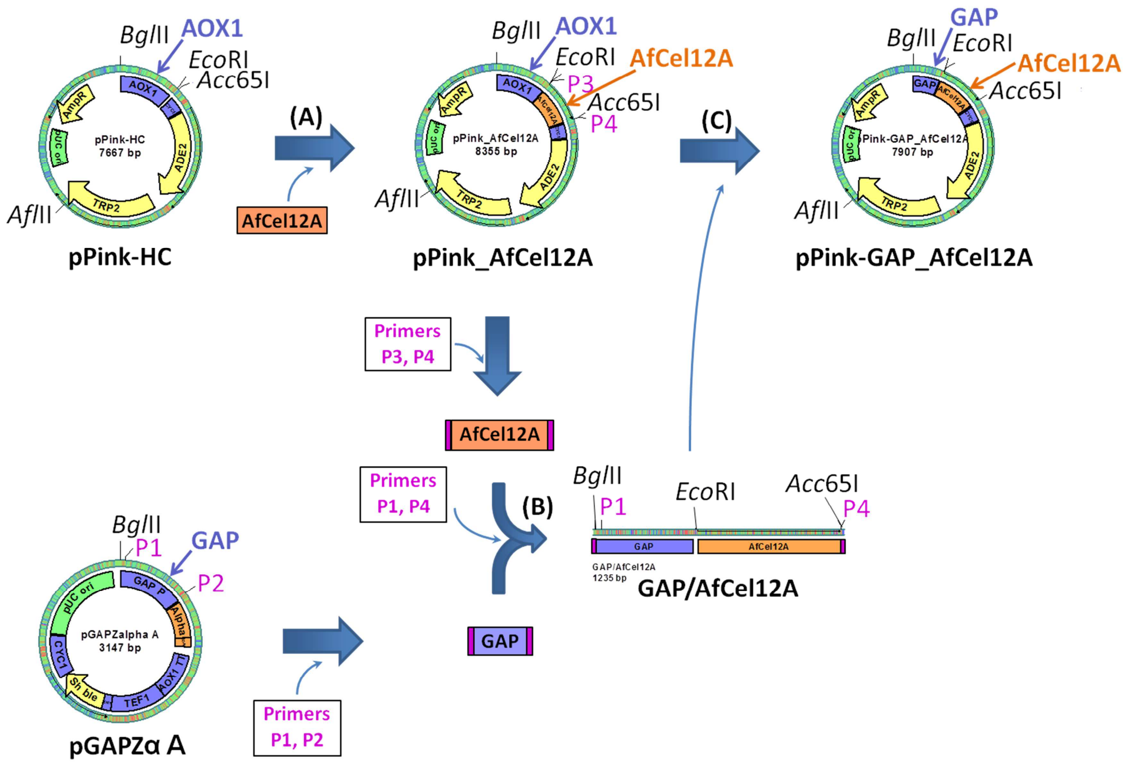

Sequences around the translation initiation site: **(D)**

```
pPink_AfCel12A        931 ATTCGAAACG GAATTCGAAA CGATGAAGAC TTTTGCTATC CT
pPink-GAP_AfCel12A    471 TTGAACAACT ATTTCGAAAC GGAATTCGAA ACGATGAAGA CT
```

Figure S1 Strategy for constructing vectors for expressing *Af*Cel12A in PichiaPink™ under the AOX1 or GAP promoters. First **(A)** a codon-optimized synthetic gene encoding *Af*Cel12A (see Methods) was inserted into the multiple-cloning site of pPink-HC (yielding pPink_AfCel12A); then **(B)** the GAP promoter was amplified from pGAPZαA vector and fused to the *Af*Cel12A gene amplified from the pPink_AfCel12A construct (GAP/AfCel12A); finally **(C)** the AOX1/AfCel12A fragment in the pPink_AfCel12A construct was replaced by the GAP/AfCel12A fragment, yielding pPink-GAP_AfCel12A. The sites of restriction enzymes and primers used are marked on the plasmid maps. Insert **(D)** shows the sequence of pPink-AfCel12A and pPink-GAP_AfCel12A around the translation initiation site; part of the promoters are indicated in red, the *Eco*RI restriction site in italic, the Kozak sequence in bold and the start codon is underlined.

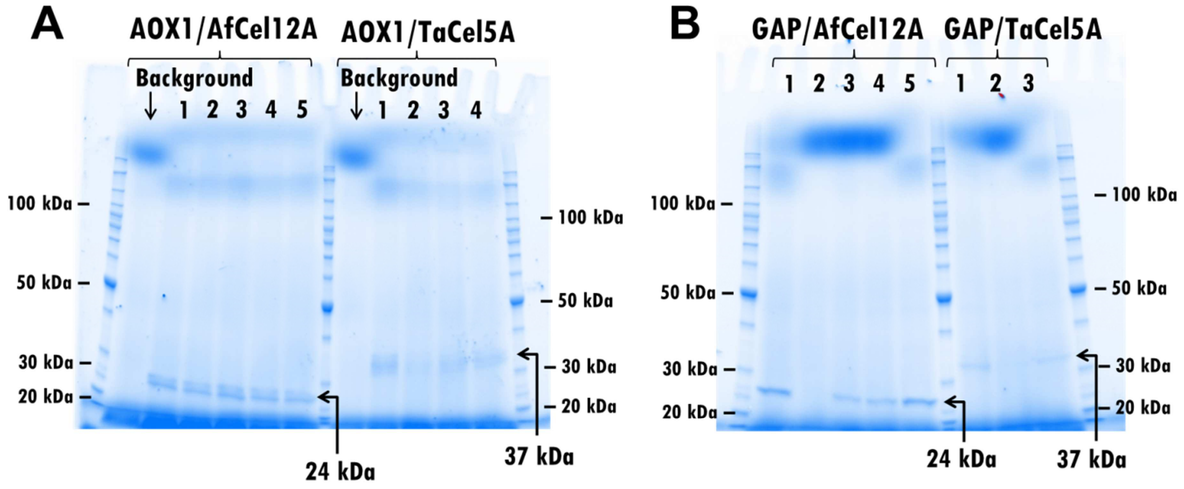

Figure S2 SDS-PAGE of culture supernatants from tube-scale screening experiments. Culture supernatants from AOX1-transformants **(A)** were harvested 48 hours after induction with methanol (BMMY medium); culture supernatants of GAP-transformants **(B)** grown on glucose (YPD medium) were harvested after 48 hours. For more details see Methods. Clones are indicated by a number above the lanes. The expected molecular weights of the secreted endoglucanases (24 kDa for *Af*Cel12A and 37 kDa from *Ta*Cel5A) are marked with arrows. Protein standard was the Benchmark Protein Ladder (Invitrogen). Samples labeled “background” show culture supernatants of AOX1/AfCel12A clone 1 and AOX1/TaCel5A clone 1 harvested after 48-hours of growth on glycerol (BMGY medium) without any induction.

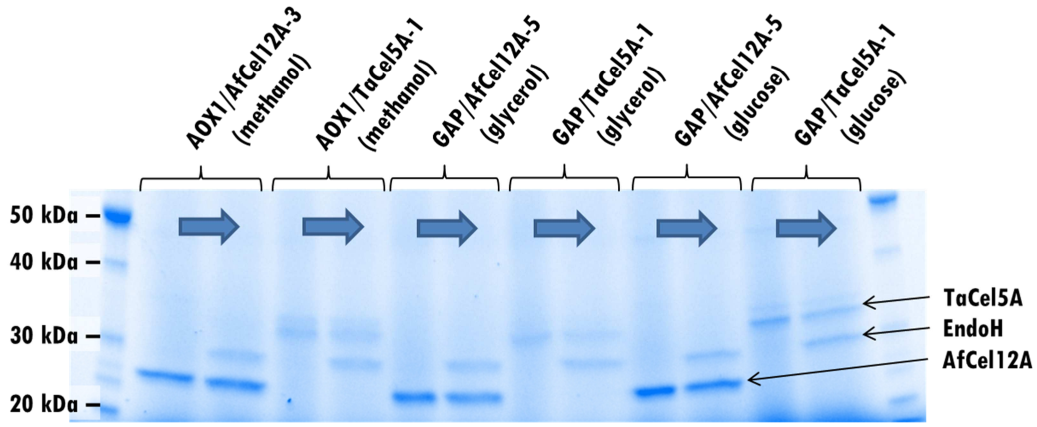

Figure S3 EndoH treatment of supernatants. The clones and carbon source are marked above the wells; blue arrows indicate EndoH treatment. The positions of Endo-H, *Af*Cel12A and *Ta*Cel5A are indicated.

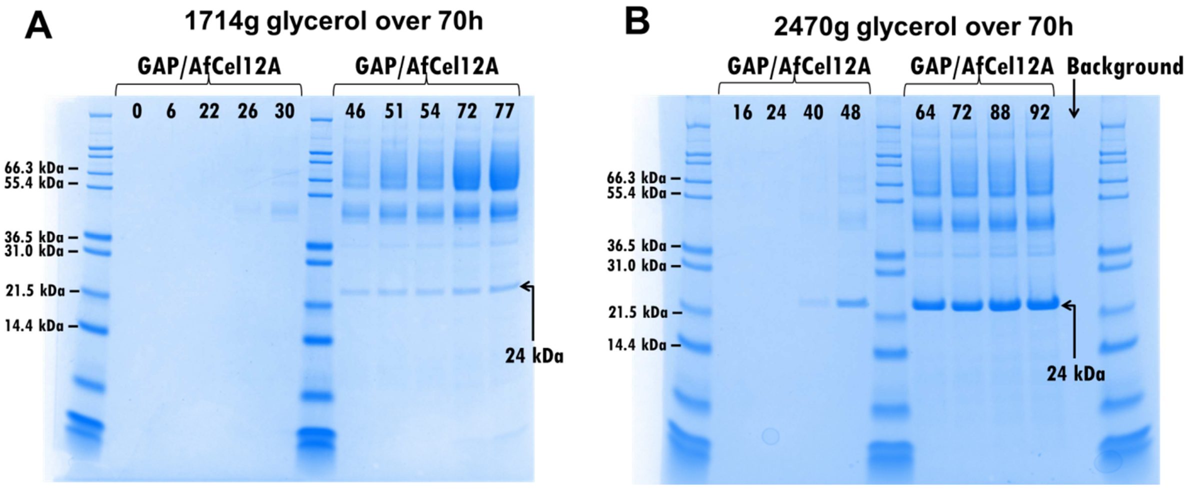

Figure S4 Improving *Af*Cel12A expression by increasing the carbon load during glycerol-fed fermentation. Fermentations of *Af*Cel12A were run in batch mode for 24 h, followed by glycerol feed as indicated above the gel images (see Methods for more details). Culture supernatant samples were collected during the fermentation and run on SDS-PAGE. Sample lanes are marked with the age of the culture in hours. The band marked as 24 kDa represents *Af*Cel12A. The protein standard was Mark12 Unstained Standard (Invitrogen).
